# Psychosocial Determinants Among Hospital and Primary Healthcare Professionals Towards Cancer and Cancer Patients in Croatia

**DOI:** 10.3390/jcm15072804

**Published:** 2026-04-07

**Authors:** Darko Kotromanovic, Ivana Kotromanovic Simic, Nika Lovrincevic Pavlovic, Marija Olujic, Sebastijan Spajic, Luka Peric, Tara Cvijic Peric, Matea Matic Licanin, Ilijan Tomas, Ivan Miskulin

**Affiliations:** 1 Faculty of Medicine Osijek, Josip Juraj Strossmayer University of Osijek, 31000 Osijek, Croatia; kotromanovic93@gmail.com (D.K.); ivsimic@mefos.hr (I.K.S.); nika.felicita@gmail.com (N.L.P.); molujic9@gmail.com (M.O.); sebastijanspajic@gmail.com (S.S.); lukaperic2@gmail.com (L.P.); tara.cvijic95@gmail.com (T.C.P.); matic.ma@gmail.com (M.M.L.); 2 Oncology Clinic, University Hospital Centre Osijek, 31000 Osijek, Croatia; 3 Ophthalmology Polyclinic Dr. Balog, 31000 Osijek, Croatia

**Keywords:** Croatia, emotions, empathy, healthcare professionals, neoplasms, patients, social stigma

## Abstract

**Background/Objectives:** Cancer places emotional and psychosocial demands on healthcare professionals; therefore, this study aimed to examine sociodemographic and psychosocial determinants, including emotional competence, empathy, and stigma, and to assess their interrelationships with mental health, attitudes towards cancer, and cancer-related stigma among healthcare professionals involved in cancer care. **Methods:** This cross-sectional study was conducted from July 2025 to January 2026 via a self-administered questionnaire among 264 hospital and primary care healthcare professionals in Osijek, Croatia (69 men and 195 women; median age 37 years, IQR 31–47, age range 20–64 years), all directly involved in providing healthcare to cancer patients in Croatia. **Results:** Significant differences were observed by gender, age, occupation, and workplace. Men were more frequently physicians and had higher education levels and socioeconomic status, whereas women achieved higher scores in emotional competence and empathy. Physicians more often had shorter overall work experience and reported greater perceived controllability of cancer. Age-related differences were found in perceived discrimination, stigma, and controllability of cancer. Primary healthcare professionals showed a higher level of empathy and proactivity and a lower perception of cancer as an incurable disease. Higher empathy was associated with lower stigma, while negative emotions and greater proactivity were associated with higher stigma, and emotional competence was a strong predictor of empathy. **Conclusions:** The study identified notable sociodemographic and psychosocial differences among healthcare professionals. Emotional competence strongly predicted empathy, which was inversely associated with cancer-related stigma, suggesting potential targets for interventions to improve attitudes towards cancer care. Furthermore, women exhibited significantly higher emotional competence and empathy than men, highlighting the importance of incorporating gender-specific perspectives into developing educational and support strategies for cancer healthcare professionals.

## 1. Introduction

Cancer is one of the leading public health challenges of the 21st century, with a continuous rise in new diagnoses and highly complex psychosocial consequences for patients, their families, and the healthcare professionals who care for them [1]. Patient-centred communication is positively associated with health-related quality of life and lower symptom burden in cancer patients. Meanwhile, the quality of relationships with physicians directly increases perceived healthcare quality [2]. Caring for cancer patients requires both technical and psychosocial skills, with communication skills—such as empathic response, active listening, and the ability to provide information—being particularly essential [3]. Therefore, understanding the psychosocial factors that shape healthcare professionals’ perceptions and behaviours towards cancer and cancer patients is critical for improving the quality of care.

Cancer-related stigma is a prominent psychosocial phenomenon that negatively affects the course of illness and health outcomes, and poses a particular challenge when present among healthcare professionals. Akin-Odanye and Husman, in a systematic review, reported that cancer stigma was expressed across various segments of society, including elites and healthcare providers, and that developing countries reported higher levels of stigma, with experiences varying by cancer type. Cancer was consistently perceived as a death sentence, which encouraged patients to conceal their diagnosis, delay help-seeking, and reduce cooperation with physicians. This perception was predominantly associated with negative psychosocial outcomes, although some patients also reported elements of post-traumatic growth [4]. Another review emphasises that perceiving cancer as a fatal disease has far-reaching negative consequences for health behaviours and outcomes. It highlights that stigma is not only an individual phenomenon but also a structural issue within healthcare systems. Along with global evidence that cancer-related stigma significantly affects cancer incidence, disease burden, and survival outcomes, these findings underscore that identifying, measuring, and reducing cancer stigma are crucial for lowering the global cancer burden and advancing health equity [5]. However, despite the importance of reducing stigmatising behaviour, there remains a paucity of the literature on interventions targeting cancer-related stigma.

It is particularly concerning when stigmatising attitudes are held by healthcare professionals themselves, as they then become a barrier to quality care rather than its enablers. Research on healthcare professionals’ attitudes towards cancer points to the presence of negative perceptions and fatalistic beliefs even within the professional community. In 2003, Kearney et al. empirically documented negative attitudes among oncology healthcare professionals, highlighting the need for educational programmes and support systems to change those attitudes [6]. One recent study describes fatalism and nihilism as widespread attitudes among clinicians dealing with lung cancer, noting that such attitudes reduce motivation for aggressive treatment and proactive preventive action. Perceptions of patient self-blame, discomfort in communicating with patients, and the belief in the inevitability of a fatal outcome have been identified as dimensions of stigma that healthcare professionals may internalise as a result of daily exposure to the most severe aspects of oncological illness [7].

Empathy is consistently recognised in the literature as a key protective factor that reduces stigmatising attitudes and improves the quality of care for cancer patients. In a study with nursing students, Arda Sürücü demonstrates that higher levels of empathy directly correlate with fewer negative perceptions of cancer patients and recommends introducing empathy-focused education into healthcare curricula [8]. Furthermore, higher physician empathy was associated with lower perceived stigma and anxiety, and greater self-efficacy in patients, which, in turn, relates to better psychological functioning in those affected [9]. A meta-analysis further confirms that physician empathy has statistically significant positive effects on cancer patient outcomes, emphasising the importance of systematically developing empathic competencies in oncological practice [10]. Williamson et al. highlight that stigma acts as a barrier to patients opening up to their physicians, and that an empathic approach by the physician is essential for overcoming this barrier and establishing a therapeutic alliance. Compassion not only humanises the physician–patient relationship, but also actively reduces prejudice and discriminatory behaviour [11]. The foundation of empathic competencies in healthcare professions is increasingly recognised in the construct of emotional intelligence, or emotional competence—the ability to recognise, understand, manage, and express emotions. Birks and Watt were early advocates of emotional intelligence as a key competency for communication with patients, and discussed ways of measuring and developing it within healthcare professions [12]. Emotional intelligence training has frequently been found to lead to improvements in healthcare professionals’ emotional intelligence, with some primary data also indicating potential benefits for stress reduction and improved patient-centred approaches [13].

In addition to these findings, gender differences in emotional intelligence are well-documented. Deng et al. [14] found that women have higher overall levels of emotional intelligence than men, while Kitsios et al. [15] further demonstrate that these differences are more pronounced in the dimensions of emotional perception and regulation. These findings suggest that gender may be a relevant factor when planning educational and organisational interventions within the healthcare system.

Despite growing interest in the psychosocial aspects of oncological care, the available literature reveals a lack of studies that simultaneously examine multiple psychosocial determinants in healthcare professionals—emotional competence, mental health, empathy in palliative care, attitudes towards cancer, and perceived stigma—and link them to demographic and professional characteristics such as gender, age, and workplace setting. Comparative studies of healthcare professionals working in hospital settings versus those in primary healthcare are particularly scarce, even though differences in working environment, daily exposure to the most severe disease outcomes, and access to palliative care may significantly shape their attitudes, empathic capacity, and propensity for stigmatisation. Understanding these differences is fundamentally important for designing targeted educational programmes and interventions to enhance the psychosocial competencies of healthcare professionals.

Moreover, the majority of existing studies on psychosocial determinants in oncology settings have focused exclusively on physicians and nurses. Meanwhile, other professional groups, such as medical physicists, radiologic technologists, clinical psychologists, and physiotherapists, have been largely overlooked, despite their direct and daily involvement in cancer care [3,8,16,17]. Each of these professional groups operates within a distinct role, with varying degrees of patient contact, emotional load, and proximity to cancer outcomes. For example, radiologic technologists and physicists regularly perform diagnostic and treatment procedures on cancer patients but rarely appear in psychosocial research. Meanwhile, physiotherapists and clinical psychologists interact with patients across prolonged periods of recovery. This professional diversity likely shapes attitudes, empathic responses, and stigma in ways that cannot be captured by studying physicians and nurses alone. Restricting samples to these two groups, therefore, risks producing an incomplete picture of the psychosocial climate within diverse healthcare professionals involved in cancer care. To the best of our knowledge, this is the first study in Croatia to include this broader spectrum of healthcare professionals. Our work enables a more comprehensive assessment of psychosocial determinants among the diverse healthcare professionals involved in cancer care and provides a foundation for designing targeted, profession-specific educational interventions.

This study aimed to examine sociodemographic and psychosocial determinants among hospital and primary care healthcare professionals in Osijek, Croatia, in relation to cancer and cancer patients, and to assess the interrelationships between emotional competence, mental health, empathy, attitudes towards cancer, and cancer-related stigma.

## 2. Materials and Methods

### 2.1. Study Design

This study was designed as a cross-sectional study, conducted between July 2025 and January 2026 among healthcare professionals in Osijek, Croatia.

### 2.2. Study Setting and Participants

In this study, we examined psychosocial determinants among hospital and primary healthcare professionals regarding cancer and cancer patients who were employed at the time of the study at the University Hospital Centre Osijek (UHC Osijek) or the Osijek-Baranja County Health Centre (OBC HC). Regarding the sampling strategy, we used a convenience-based sample consisting of healthcare professionals employed at selected clinics and clinical departments of the UHC Osijek (tertiary level) and at selected departments of the OBC HC (primary level). The UHC Osijek clinics and clinical departments included in the study were: Clinic of Surgery, Clinic of Gynaecology and Obstetrics, Clinic of Internal Medicine, Clinic of Psychiatry, Clinic of Neurology, Clinic of Oncology, Clinic of Infectious Diseases, Clinic of Ophthalmology, Clinic of Otorhinolaryngology and Head and Neck Surgery, Clinic of Neurosurgery, Clinic of Orthopaedics and Traumatology, Clinical Department of Nuclear Medicine and Radiation Protection, Clinical Department of Diagnostic and Interventional Radiology, Department of Urology, Department of Maxillofacial and Oral Surgery, and Department of Dermatology and Venereology. For healthcare professionals employed at OBC HC, the study included those working in general medicine practices, a mobile palliative care team, and community health nursing teams. Regarding occupation, the study included physicians, nurses/technicians, physicists, radiologic technologists, clinical psychologists, and physiotherapists. Healthcare professionals listed above were approached through personal contacts with the authors, either verbally or in writing, and were included based on their direct involvement, to varying degrees, in the healthcare of cancer patients in Croatia.

The inclusion criteria for participation were: providing informed consent to participate, being aged 18 years or older, having a medical education, and current employment at the selected clinics and clinical departments of the UHC Osijek or at the selected departments of the OBC HC.

Exclusion criteria for participation were refusal to participate in the study, a diagnosed mental disorder (either based on self-report prior to completing the questionnaire or on the researchers’ prior knowledge of a confirmed diagnosis), being underage (younger than 18 years), non-medical education, retirement, and current non-employment at UHC Osijek or the OBC HC.

Before starting this study, based on calculations obtained using the G*Power test (Heinrich Heine University, Düsseldorf, Germany, version 3.1.9.6), we determined that it was necessary to include at least 124 participants. Therefore, our research included 264 participants. The response rate varied across workplaces (selected clinics and clinical departments of the UHC Osijek and selected departments of the OBC HC), as well as across occupations; therefore, it was not possible to determine a single overall response rate for the study.

### 2.3. Measurements

The questionnaire used in this study comprised the following sections: questions on sociodemographic characteristics (age, sex, place of residence, current partnership status, number of household members, socioeconomic status, current work activity, highest completed level of education, occupation, workplace, years of work experience in the healthcare system, years of work experience in the healthcare system with cancer patients, previous contact [“Have you heard of…?”] with the National Cancer Early Detection Programme, previous attendance at screening appointments within the National Cancer Early Detection Programme, and previous performance of self-examinations, such as of the breasts, skin, or testicles); a simple analogue scale (1–10) for subjective assessment of quality of life [18]; and validated instruments (distributed in Croatian and validated in prior studies conducted in Croatia and among the Croatian population): the Emotional Competence Questionnaire (UEK-45) [19]; the Mental Health Inventory-5 (MHI-5) [20,21]; the Empathy in Palliative Care Scale [20]; the Questionnaire of Attitudes in Cancer [22,23]; and the Cancer-related Perceived Stigma Questionnaire version 1.0 [22,24]. Some components of the questionnaire were not original and consisted of previously developed and validated instruments (UEK-45, MHI-5, Empathy in Palliative Care Scale, Questionnaire of Attitudes in Cancer, and Cancer-related Perceived Stigma Questionnaire), all of which had been translated into Croatian and validated in studies among the Croatian population [18,19,20,21,22,23,24]. In contrast, the questions assessing participants’ sociodemographic characteristics were original and designed for this study. The complete questionnaire was developed specifically for this purpose and is available from the authors upon request.

Emotional competence was assessed using the UEK-45 questionnaire [19], which includes three subscales: the ability to perceive and understand emotions (Cronbach’s alpha = 0.948), the ability to express and label emotions (Cronbach’s alpha = 0.882), and the ability to regulate and manage emotions (Cronbach’s alpha = 0.914). Higher scores indicate a higher level of emotional competence. A total emotional competence score was calculated from these subscales, with an internal consistency coefficient (Cronbach’s alpha) of 0.966. Participants’ mental health was assessed using a Croatian version [20] of the original Mental Health Inventory-5 (MHI-5) [21], a five-item self-report questionnaire covering emotional well-being and symptoms of anxiety and depression. The internal consistency of the MHI-5, as measured by Cronbach’s alpha, was 0.841. Higher scores indicate better mental health. Empathy in palliative care was assessed with a 14-item questionnaire [20], where higher scores indicate a higher level of empathy in palliative care. The internal consistency coefficient of this questionnaire, Cronbach’s alpha, was 0.944. Attitudes towards cancer were assessed using a Croatian version [22] of the original Questionnaire of Attitudes in Cancer [23], a self-report questionnaire comprising three domains: perceived controllability of cancer (Cronbach’s alpha = 0.704), negative emotional responses (Cronbach’s alpha = 0.948), and intention for proactive behaviour in the context of cancer (Cronbach’s alpha = 0.879). Higher scores on the Controllability of Cancer subscale indicate a belief in greater personal influence over the likelihood of developing cancer. Higher scores on the Negative Emotional Responses subscale indicate more pronounced negative emotions related to cancer. On the Intention for Proactive Behaviour subscale, higher scores indicate greater motivation for proactive behaviour related to cancer and cancer patients. Cancer-related stigma was assessed using a Croatian version [22] of the original Cancer-related Perceived Stigma Questionnaire version 1.0 [24], a 12-item self-report questionnaire comprising three domains: Impossibility of Curing Cancer, Stereotypes and Discrimination, and Overall Cancer-related Stigma. Scores closer to the upper end of the scale indicate more pronounced stigma, while lower scores reflect less stigmatising attitudes. The internal consistency coefficient for the entire questionnaire, Cronbach’s alpha, was 0.790.

### 2.4. Ethical Procedures

This study was approved by the Ethics Committee of the University Hospital Centre Osijek (approval date: 3 June 2025, Reg. No.: R1-6275/2025), the Ethics Committee of the Osijek-Baranja County Health Centre (approval date: 2 July 2025, Reg. No.: 03-1744-1/25), and the Ethics Committee of the Faculty of Medicine Osijek (approval date: 15 July 2025, Class: 602-04/25-08/7, Reg. No.: 2158-61-46-25-166).

### 2.5. Statistical Analysis

Categorical variables are presented as absolute and relative frequencies. Differences between categorical variables were assessed using the chi-square test or Fisher’s exact test, as appropriate. The normality of continuous variables was evaluated using the Shapiro–Wilk test. Continuous variables are described using the median and interquartile range (IQR). Differences in continuous variables between two independent groups were analysed using the Mann–Whitney U test, with the Hodges–Lehmann median difference and corresponding 95% confidence interval reported. Comparisons among three or more independent groups were performed using the Kruskal–Wallis test, followed by post hoc Conover tests where appropriate. Associations between variables were assessed by Spearman’s correlation coefficient, and correlation matrices were graphically displayed as heat maps to facilitate interpretation of the strength and direction of the relationship. Multivariate linear regression analyses were conducted and adjusted for sex, age, profession, and workplace. Psychosocial variables were included using a stepwise selection procedure to identify the most relevant predictors and minimise the risk of overfitting given the sample size. The primary assumptions of linear regression, including linearity, normality of residuals, homoscedasticity, and multicollinearity, were assessed prior to analysis, and no major violations were observed. All *p*-values were two-sided, and the level of statistical significance was set at α = 0.05.

Statistical analyses were performed using MedCalc^®^ Statistical Software (MedCalc Software Ltd., Ostend, Belgium, version 23.2.8.) and SPSS (IBM Corp, Armonk, NY, USA, version 23) [25]. The study was reported in accordance with the STROBE guidelines for cross-sectional studies [26,27].

## 3. Results

### 3.1. Sociodemographic Characteristics of the Participants

The study included 264 respondents, all healthcare professionals, of whom 195 (73.9%) were women. Gender distribution differed significantly by occupation and workplace, with a higher proportion of men among physicians and those employed at UHC Osijek (Chi-square test, *p* < 0.001). The median age of respondents was 37 years (interquartile range 31 to 47), with ages ranging from 20 to 64 years. The number of household members ranged from one to six. Physicians were significantly more likely to have a graduate or MSc/PhD level of education compared with other healthcare professionals (Chi-square test, *p* < 0.001). Household socioeconomic status also differed by occupation (*p* < 0.001), with above-average status more frequently reported among physicians (Table 1).

Significant differences in total work experience in the healthcare system were observed by occupation (Chi-square test, *p* = 0.001), with physicians more often having shorter work experience (up to 10 years), while other healthcare professionals more frequently belonged to groups with longer work experience. In contrast, years of working with cancer patients did not differ significantly by occupation or workplace. All respondents were familiar with the National Cancer Early Detection Programmes. Attendance at screening appointments within these national programmes, as well as the frequency of performing self-examinations, did not differ significantly between the groups observed.

Participants subjectively assessed their quality of life on an analogue scale from 1 (not at all satisfied) to 10 (completely satisfied). The median score was 9 (interquartile range 8 to 9), with scores ranging from 3 to 10. No significant differences in quality of life scores were found with respect to gender, age, occupation, or workplace.

### 3.2. Psychosocial Characteristics by Gender/Age/Occupation/Workplace

Psychosocial characteristics were assessed using validated questionnaires measuring emotional competence (UEK-45), mental health (MHI-5), empathy in palliative care, cancer-related stigma, and attitudes towards cancer. Women achieved significantly higher emotional competence scores than men, including the ability to perceive and understand emotions (Mann–Whitney U test, *p* = 0.003), the expression and labelling of emotions (*p* < 0.001), and the overall emotional competence score (*p* = 0.003). Women also had a higher level of empathy in palliative care (*p* = 0.002) and more pronounced negative emotional responses towards cancer (*p* = 0.04), whereas men showed a higher level of perceived controllability of cancer (*p* = 0.008). No significant gender differences were found in emotion regulation, overall mental health (according to MHI-5), perceived cancer-related stigma, or intention for proactive behaviour (Table 2).

Significant differences were found in the perception of cancer-related discrimination across age groups (Kruskal–Wallis test, *p* = 0.004), with respondents aged 41 to 50 years reporting a higher level of perceived discrimination than younger participants. Differences were also observed in the overall perception of cancer-related stigma (Kruskal–Wallis test, *p* = 0.03), with higher scores in the middle-age groups compared to the youngest group. Age-related differences in perceived controllability of cancer were significant (Kruskal–Wallis test, *p* = 0.04), with respondents up to 30 years of age reporting a higher level of perceived cancer controllability than older age groups. No significant differences were found for other domains of emotional competence, mental health, empathy in palliative care, stereotypes, negative emotional responses, or intention for proactive behaviour (Table 3).

No significant differences were found between physicians and other healthcare professionals in emotional competence (UEK-45), overall mental health (MHI-5), empathy in palliative care, or perceived cancer-related stigma. The only significant difference was in perceived controllability of cancer: physicians reported a higher level of perceived controllability, indicating a stronger belief that cancer can be influenced (Mann–Whitney U test, *p* = 0.007).

Healthcare professionals employed at OBC HC achieved significantly higher empathy scores in palliative care than those working at UHC Osijek (Mann–Whitney U test, *p* < 0.001). They also reported a lower perception of the impossibility of curing cancer (Mann–Whitney U test, *p* = 0.01); that is, they were less likely to view cancer as an inevitably incurable disease, and reported a higher intention for proactive behaviour related to cancer (Mann–Whitney U test, *p* = 0.02). No significant differences by workplace were found in other domains of emotional competence, overall mental health, cancer-related stigma, or attitudes towards cancer (Table 4).

### 3.3. Correlations Between Psychosocial Variables

Using Spearman’s correlation coefficient, significant, mostly weak to moderate associations were observed between the examined psychosocial characteristics, with similar patterns of association across all workplace groups (Table 5).

**Figure 1:**
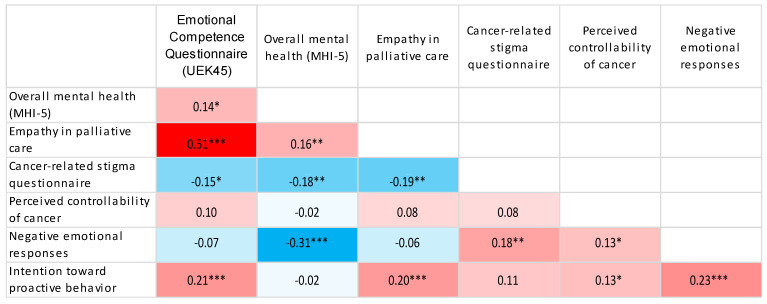
Heatmap of Spearman correlation coefficients between study variables. Red indicates positive correlations, blue indicates negative correlations, and colour intensity reflects the strength of the association. * *p*-value < 0.05; ** *p*-value < 0.01; *** *p*-value < 0.001.

**Figure 2:**
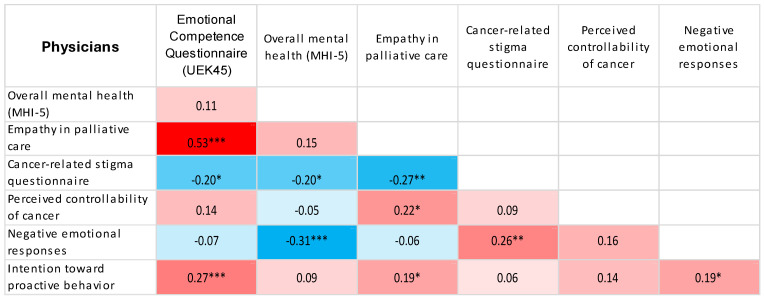
Heat map displaying Spearman correlation coefficients between study variables for physicians. Red denotes positive correlations, blue denotes negative correlations, and colour intensity represents the magnitude of the association. * *p* < 0.05; ** *p* < 0.01; *** *p* < 0.001.

**Figure 3:**
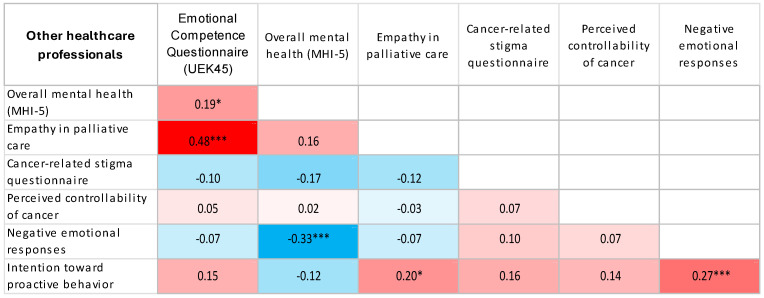
Heat map displaying Spearman correlation coefficients between study variables for other healthcare professionals. Red denotes positive correlations, blue denotes negative correlations, and colour intensity represents the magnitude of the association. * *p* < 0.05; *** *p* < 0.001.

**Figure 4:**
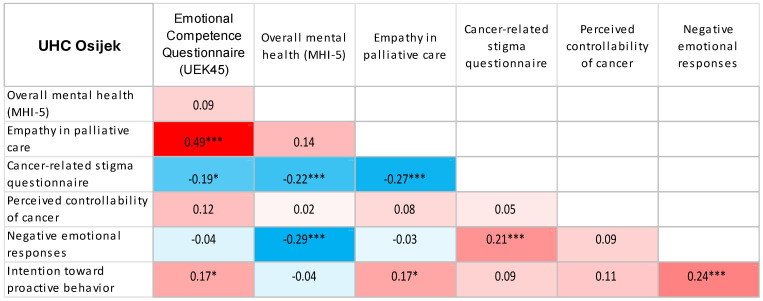
Heat map displaying Spearman correlation coefficients between study variables among UHC Osijek healthcare professionals. Red denotes positive correlations, blue denotes negative correlations, and colour intensity represents the magnitude of the association. * *p* < 0.05; *** *p* < 0.001.

**Figure 5:**
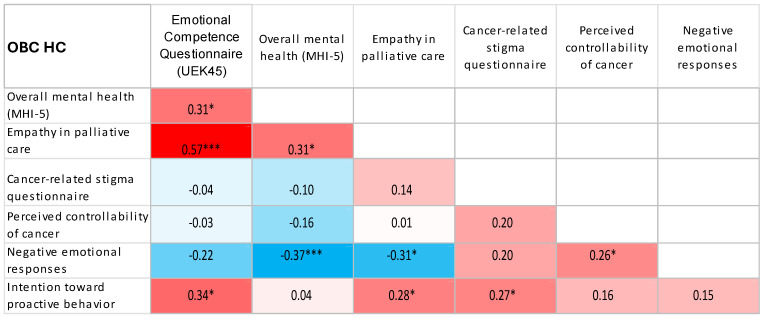
Heat map displaying Spearman correlation coefficients between study variables among OBC HC healthcare professionals. Red denotes positive correlations, blue denotes negative correlations, and colour intensity represents the magnitude of the association. * *p* < 0.05; *** *p* < 0.001.

Using heat maps, we presented the correlations between the study variables as determined by Spearman correlation coefficients (Figure 1, Figure 2, Figure 3, Figure 4 and Figure 5).

### 3.4. Multivariate Linear Regression Analyses

Multivariate linear regression analyses were conducted with cancer-related stigma and empathy in palliative care as dependent variables. Gender, age, occupation, and workplace were entered into the models using the enter method, while psychosocial questionnaire results were included using a stepwise selection procedure. In the adjusted model, higher empathy in palliative care was significantly associated with lower cancer-related stigma (β = −0.094), whereas stronger negative emotional responses (β = 0.084) and greater intention for proactive behaviour (β = 0.096) were associated with higher stigma scores. The model explained 11.2% of the variance and showed a small to moderate effect size (Cohen’s f^2^ = 0.13). Emotional competence (UEK-45) was positively associated with empathy in palliative care (β = 0.52), explaining 28% of the variance in the adjusted model with a large overall effect size (Cohen’s f^2^ = 0.39) (Table 6).

## 4. Discussion

This study examined psychosocial determinants among healthcare professionals employed at UHC Osijek and OBC HC who provide healthcare to cancer patients in Croatia. Women demonstrated higher emotional competence and empathy, while men and physicians reported higher perceived controllability of cancer. Primary healthcare professionals reported greater empathy and stronger intentions for proactive behaviour, as well as a lower tendency to view cancer as an incurable disease.

Emotional competence was a key predictor of empathy, while higher empathy was associated with lower cancer-related stigma. Emotional intelligence, i.e., competence, is defined as the ability to identify and recognise the meaning and concepts of emotions. It can be defined as a function of labelling emotions appropriately, using them as a guide for behaviour, adapting to situations, and reaching goals based on emotional information [14]. Several studies [14,15,28,29] among healthcare professionals have reported higher levels of emotional intelligence in women than in men, particularly in domains related to emotional awareness and the evaluation of one’s own emotions, indicating that women healthcare professionals may have an advantage in emotion-related skills. Our results also support this finding: women achieved significantly higher emotional competence scores than men, including the ability to perceive and understand emotions, the expression and labelling of emotions, and the overall emotional competence score.

Regarding empathy, results from various studies [30,31,32,33,34] suggest that female healthcare workers tend to display higher levels of empathy and are more likely to use empathic communication strategies, which is particularly important in emotionally demanding contexts such as cancer care. Although empathy is equally important among all healthcare workers, regardless of whether they work in primary or hospital settings, some studies [30,35,36] indicate that differences still exist. Research indicates that residents in people-oriented specialties, such as general medicine, internal medicine, obstetrics and gynaecology, emergency medicine, paediatrics and psychiatry, exhibit higher levels of empathy than those in technology-oriented specialties, such as anaesthesiology, neurosurgery, surgery, and radiology. Physicians in people-oriented specialties have more opportunities for direct and repeated patient contact and more extensive communication, which substantially contributes to the development of positive interpersonal relationships, a better understanding of patients’ psychosocial context, and consequently, higher levels of empathic engagement [37,38,39,40,41]. In our study, primary care physicians had higher empathy scores, which is consistent with these findings and may reflect the cumulative effect of long-term, trust-based relationships and longitudinal monitoring of patients’ health over time. According to a study by Pedersen et al. [36], in primary healthcare, higher physician empathy has been linked to more effective use of clinical “gut feeling” in cancer diagnosis and, consequently, to better patient–physician relationships. A study by Derksen et al. [35] showed the importance and effectiveness of empathy in general medical practice and primary healthcare, as long-term relationships and continuity enable better recognition of changes, earlier detection of cancer symptoms, and greater adherence to the diagnostic process. Moreover, in hospital oncology settings, healthcare workers’ empathic responses to patients’ emotions remain relatively infrequent despite their clear benefits for psychological adjustment. A study by Lelorain et al. [10] showed that physicians’ empathy was associated with favourable patient outcomes, which is especially important in cancer patients. These results are consistent with those obtained in our study, which showed that female healthcare workers are more empathetic than their male colleagues, and that primary healthcare workers, regardless of gender, are more empathetic than their hospital colleagues. The organisation of primary healthcare in Croatia aligns with this model, as patients are free to choose their own primary healthcare physician—whether a general physician, gynaecologist, paediatrician, or another primary healthcare provider—according to their personal preferences and consult these selected physicians for most of their healthcare needs. This structure enables patients and their physicians to develop close, enduring relationships through the continuous monitoring and management of patients’ health needs [42] and may partly explain why primary healthcare professionals in our sample reported higher empathy levels compared with physicians in other specialties. In addition, cultural expectations in the Croatian healthcare system, where primary care physicians are often perceived as “family doctors” responsible not only for solving medical problems but also for providing emotional support and guidance, likely further reinforce the development and expression of empathy in primary care settings. To the best of our knowledge, there are currently no data on gender differences in emotional intelligence or empathy among health professionals in Croatia, regardless of their work settings. However, a few studies conducted among Croatian students [43,44] have found that women have higher emotional intelligence and empathy scores than men, which is consistent with our findings.

Previous research indicates that medical students’ empathy changes during their education, with levels tending to decline as training progresses. Additionally, empathy levels appear to influence specialty choice, highlighting the need for further interventions in medical education to foster and preserve empathy in future physicians and other healthcare professionals [37,45,46,47,48,49]. The results from the study by Kearney et al. [6] showed overall negative attitudes towards cancer among healthcare professionals, regardless of gender, occupation, or clinical experience. In contrast, some studies reported overall positive attitudes towards cancer among healthcare professionals. A study conducted in Turkey among nurses [16] found that nurses generally do not have negative attitudes towards cancer, regardless of age, gender, marital status, or education level. Another study conducted among physicians [17] showed that, in general, physicians have a positive overall attitude towards cancer and cancer patients, regardless of their gender. In our sample, female healthcare professionals exhibited more intense negative emotional responses to cancer, while men reported a higher level of perceived controllability of cancer. These findings suggest that different groups of healthcare professionals may emphasise different aspects of their attitudes towards cancer, ranging from heightened emotional concern to greater perceived control. This aligns with previous studies reporting both more negative and more positive attitudes among healthcare professionals.

In our study, physicians reported higher perceived controllability of cancer compared with other healthcare professionals, suggesting greater therapeutic optimism among physicians. This finding is consistent with the study by Loh et al. [50], which demonstrated that beliefs about the curability and controllability of cancer often differ between oncologists and other stakeholders who provide care to cancer patients. These data indicate that even within oncology, different professional and lay groups can hold markedly different expectations about the potential for controlling or curing cancer.

Perceiving cancer as a controllable disease may also serve as a cognitive counterbalance to fatalistic and nihilistic attitudes towards cancer. The American Cancer Society’s National Lung Cancer Roundtable has emphasised that stigma and therapeutic nihilism in lung cancer can undermine prevention, early detection, treatment, and survivorship efforts, highlighting the need to shift the narrative away from inevitability and hopelessness [7]. From this perspective, the higher perceived controllability of cancer found in our study among physicians and younger healthcare professionals could represent a potentially protective mindset that counteracts fatalism and may promote more active engagement in prevention, treatment, and follow-up.

In Croatia, a study conducted among students [22] found that they had a high level of perceived controllability of cancer and a high level of negative emotions related to cancer. This is consistent with our research, which showed that younger age groups of respondents have a greater perceived controllability of cancer.

Some studies also suggest a connection between empathy and attitudes or stigma towards cancer patients. A study conducted among nursing students in Turkey showed that their negative perception of cancer decreased as their empathic skills increased [8]. Two studies conducted by Yang et al. found that physicians’ empathy was negatively associated with patients’ stigma and anxiety, and positively associated with patients’ self-efficacy. In these studies, higher levels of empathy were linked to better psychological outcomes and, indirectly, to more favourable biological outcomes of the disease. Patients’ stigma and self-efficacy mediated the relationship between physicians’ empathy and both psychological distress and patients’ NK cell subset, with stigma exerting a stronger mediating effect than self-efficacy [9,51]. These findings are consistent with our results, in which higher levels of empathy among healthcare professionals were also modestly but significantly associated with lower cancer-related stigma (β = −0.094, *p* = 0.01), suggesting that empathic abilities may serve as an upstream protective factor shaping more favourable attitudes towards cancer and people living with cancer.

Finally, numerous studies [14,52,53,54] have found that empathy is positively related to emotional competence, which our study also confirms, as our linear regression analyses showed that emotional competence was positively associated with empathy (β = 0.52, *p* < 0.001).

### Limitations of the Study

This study has certain limitations that may affect the reliability of its findings. Firstly, as a cross-sectional study, it cannot establish causal relationships but only identify associations between the variables examined. Secondly, despite the inclusion of 264 healthcare professionals, reliance on a relatively small, convenience-based, and non-representative sample drawn from only two healthcare institutions, along with varying response rates across workplaces and occupations, and the potential influence of socially desirable responses, may limit the generalisability and accuracy of the findings and introduce potential selection bias. Thirdly, the relatively short data collection period may have affected both the number and profile of recruited participants, potentially favouring the inclusion of individuals more inclined to participate in research due to their psychosocial characteristics, which should be considered when interpreting the results. Fourthly, the exclusive use of self-report questionnaires may limit the objectivity of the data, as responses can be influenced by individual interpretation and current emotional state. Finally, several potential confounders, including years of professional experience, education level, and exposure to oncology patients, were not included in the final regression models. Years of experience showed a strong correlation with age; therefore, age was retained to minimise multicollinearity. Additionally, years of experience varied substantially across occupations, and their effects were likely accounted for by variables already present in the model. Other variables were excluded to prevent overfitting, given the sample size. However, this may have resulted in residual confounding.

Further research using longitudinal and/or interventional study designs is therefore needed to clarify and better understand sociodemographic and psychosocial differences, as well as the relationships between these characteristics among healthcare professionals, with the aim of improving the quality of care and, consequently, the quality of life of cancer patients.

## 5. Conclusions

The study revealed significant sociodemographic and psychosocial differences among healthcare professionals involved in cancer care, reflecting the complexity of their emotional and cognitive responses to cancer. Women consistently exhibited higher emotional competence and empathy, while men reported greater perceived controllability of cancer. This gender difference highlights distinct emotional and cognitive approaches to patient healthcare and should be considered when designing training and support interventions for healthcare professionals. Age, occupation, and workplace were also associated with distinct patterns of perceived discrimination, stigma, and proactive responses towards cancer, highlighting the contextual nature of these attitudes. Emotional competence was identified as a key determinant of empathy, and empathy was inversely related to cancer-related stigma. These findings extend the existing literature by integrating multidimensional constructs—emotional competence, empathy, mental health, and stigma—within a multidisciplinary sample of oncology-related healthcare professionals. By including physicians, nurses, medical physicists, radiologic technologists, clinical psychologists, and physiotherapists, the study provides one of the most comprehensive psychosocial profiles of oncology practice in Croatia. Importantly, the results highlight that systematic training and support programmes to strengthen emotional competence and empathic capacities may be valuable strategies for reducing stigma and improving the quality of cancer care. Designing and implementing such interventions should therefore become an integral part of professional education and continuous development in oncology and broader healthcare contexts.

## Figures and Tables

**Table 1:** Basic characteristics of respondents by occupation and workplace location.

	Number (%) of Subjects			p *	Number (%) of Subjects		p *
	Total	Physicians	Other Healthcare Professionals		UHC Osijek	OBC HC	
Gender							
Male	69 (26.1)	50 (37.3)	19 (14.6)	**<0.001**	66 (32.8)	3 (4.8)	**<0.001**
Female	195 (73.9)	84 (62.7)	111 (85.4)		135 (67.2)	60 (95.2)	
Age groups							
under 30	56 (21.2)	30 (22.4)	26 (20)	0.14	42 (20.9)	14 (22.2)	0.07
31–40	110 (41.7)	63 (47)	47 (36.2)		76 (37.8)	34 (54)	
41–50	56 (21.2)	22 (16.4)	34 (26.2)		48 (23.9)	8 (12.7)	
51 and over	42 (15.9)	19 (14.2)	23 (17.7)		35 (17.4)	7 (11.1)	
Current partnership status							
Married	162 (61.4)	84 (62.7)	78 (60)	0.98 ^†^	124 (61.7)	38 (60.3)	0.40 ^†^
Domestic partnership	19 (7.2)	9 (6.7)	10 (7.7)		14 (7)	5 (7.9)	
In a relationship	42 (15.9)	20 (14.9)	22 (16.9)		29 (14.4)	13 (20.6)	
Widower	1 (0.4)	0	1 (0.8)		1 (0.5)	0	
Single	32 (12.1)	17 (12.7)	15 (11.5)		28 (13.9)	4 (6.3)	
Divorced	8 (3)	4 (3)	4 (3.1)		5 (2.5)	3 (4.8)	
Level of education							
High school education	46 (17.4)	0	46 (35.4)	**<0.001**	38 (18.9)	8 (12.7)	0.22
Undergraduate education	20 (7.6)	0	20 (15.4)		12 (6)	8 (12.7)	
Graduate education	126 (47.7)	80 (59.7)	46 (35.4)		94 (46.8)	32 (50.8)	
MSc/PhD	72 (27.3)	54 (40.3)	18 (13.8)		57 (28.4)	15 (23.8)	
Occupation							
Physician	134 (50.8)	134 (100)	0	**<0.001** ^†^	106 (52.7)	28 (44.4)	**0.01** ^†^
Nurse/Technician	104 (39.4)	0	104 (80)		69 (34.3)	35 (55.6)	
Physicist	5 (1.9)	0	5 (3.8)		5 (2.5)	0	
Radiologic Technologist	14 (5.3)	0	14 (10.8)		14 (7)	0	
Clinical Psychologist	4 (1.5)	0	4 (3.1)		4 (2)	0	
Physiotherapist	3 (1.1)	0	3 (2.3)		3 (1.5)	0	
Socioeconomic status ^§^							
Below average	2 (0.8)	1 (0.7)	1 (0.8)	**<0.001** ^†^	2 (1)	0	0.35 ^†^
Slightly below average	4 (1.5)	1 (0.7)	3 (2.3)		3 (1.5)	1 (1.6)	
Average	104 (39.4)	12 (9)	92 (70.8)		73 (36.3)	31 (49.2)	
Slightly above average	101 (38.3)	71 (53)	30 (23.1)		79 (39.3)	22 (34.9)	
Above average	53 (20.1)	49 (36.6)	4 (3.1)		44 (21.9)	9 (14.3)	
Workplace							
UHC Osijek	201 (76.1)	106 (79.1)	95 (73.1)	0.25	201 (100)	0	**-**
OBC HC	63 (23.9)	28 (20.9)	35 (26.9)		0	63 (100)	
Employment status							
Employed	258 (97.7)	131 (97.8)	127 (97.7)	>0.99 ^†^	197 (98)	61 (96.8)	0.63 ^†^
Currently on sick leave	6 (2.3)	3 (2.2)	3 (2.3)		4 (2)	2 (3.2)	

* Chi-square Test; ^†^ Fisher’s Exact Test; Bold values denotes statistical significance. ^§^ socioeconomic status is self-assessed.

**Table 2:** Differences in psychosocial characteristics of healthcare professionals by gender.

	Median (IQR)		Difference	95% CI	p * Value
	Male (n = 69)	Female (n = 195)			
The ability to perceive and understand emotions	3.59 (3–4)	3.82 (3.4–4.1)	0.29	0.12 to 0.47	**0.003**
The ability to express and label emotions	3.5 (3–3.9)	3.9 (3.4–4.4)	0.38	0.13 to 4.4	**<0.001**
The ability to regulate and manage emotions	3.9 (3.4–4.1)	3.9 (3.5–4.2)	0.10	−0.05 to 0.3	0.17
**Overall emotional competence score (UEK-45)**	3.6 (3.2–3.9)	3.9 (3.5–4.2)	0.22	0.09 to 0.38	**0.003**
**Overall mental health (MHI-5)**	22 (18–25)	22 (19–24)	0	−1 to 1	0.66
**Empathy in palliative care**	4 (3.7–4.5)	4.38 (4–4.8)	0.23	0.08 to 0.38	**0.002**
Impossibility of curing cancer	2 (1.8–2.5)	2 (1.5–2.3)	0	−0.25 to 0	0.20
Stereotypes	2.5 (2.3–2.8)	2.5 (2.3–2.8)	0	−0.25 to 0	0.21
Discrimination	1.3 (1–1.8)	1 (1–1.8)	0	0 to 0	0.21
**Overall cancer-related stigma**	2 (1.8–2.3)	1.9 (1.67–2.2)	−0.08	−0.17 to 0	0.11
**Attitudes towards cancer**					
Perceived controllability of cancer	2.6 (2.4–2.9)	2.6 (2.2–2.8)	−0.20	−0.2 to 0	**0.008**
Negative emotional response	2.7 (2–3)	3 (2.3–3)	0.14	0 to 0.43	**0.04**
Intention for proactive behaviour	2.5 (2.3–2.9)	2.8 (2.3–3)	0	−0.13 to 0.25	0.46

* Mann–Whitney U test. Bold value denotes statistical significance. Abbreviation: IQR—Interquartile Range.

**Table 3:** Differences in psychosocial characteristics of healthcare professionals by age.

	Median (IQR) by Age				p * Value
	Under 30 (n = 56)	31–40 (n = 110)	41–50 (n = 56)	51 and Over (n = 42)	
	(1)	(2)	(3)	(4)	
The ability to perceive and understand emotions	3.7 (3.3–4.1)	3.8 (3.4–4.1)	3.8 (3.2–4.2)	3.6 (2.9–4)	0.59
The ability to express and label emotions	3.8 (3.3–4)	3.9 (3.4–4.3)	3.8 (3.2–4.3)	3.7 (3–4.4)	0.40
The ability to regulate and manage emotions	3.9 (3.5–4.1)	3.9 (3.6–4.3)	3.9 (3.5–4)	3.7 (3.4–4)	0.08
**Overall emotional competence score (UEK-45)**	3.7 (3.4–4.2)	3.8 (3.5–4.2)	3.8 (3.5–4.1)	3.7 (3.3–3.9)	0.41
**Overall mental health (MHI-5)**	22 (20–24)	22 (18–24)	21 (18–23)	21.5 (18–24)	0.62
**Empathy in palliative care**	4.4 (4–4.7)	4.4 (4–4.8)	4 (3.6–4.9)	4.3 (3.9–4.7)	0.56
Impossibility of curing cancer	2 (1.5–2.3)	1.8 (1.5–2.3)	2 (1.8–2.3)	2.1 (1.8–2.3)	0.08
Stereotypes	2.5 (2.1–2.8)	2.5 (2.3–2.8)	2.5 (2.3–2.8)	2.4 (2–2.8)	0.45
Discrimination	1 (1–1.3)	1 (1–1.8)	1.5 (1–2)	1.4 (1–2)	**0.004** ^†^
**Overall cancer-related stigma**	1.8 (1.7–2.1)	1.8 (1.7–2.2)	2.1 (1.8–2.2)	2 (1.7–2.3)	**0.03** ^‡^
**Attitudes towards cancer**					
Perceived controllability of cancer	2.6 (2.4–2.8)	2.5 (2.2–2.8)	2.5 (2–2.8)	2.4 (2.2–2.7)	**0.04** ^§^
Negative emotional response	3 (2.4–3.1)	3 (2.3–3)	2.6 (2–3)	2.7 (2.1–3)	0.29
Intention for proactive behaviour	2.7 (2.4–2.9)	2.6 (2.3–3)	2.8 (2.3–3)	2.6 (2.3–2.9)	0.69

* Kruskal–Wallis test (post hoc test Conover). Bold value denotes statistical significance. Abbreviation: IQR—Interquartile Range. ^†^ at *p* < 0.05 statistically significant differences were observed between (1) vs. (3, 4); (2) vs. (3). ^‡^ at *p* < 0.05 statistically significant differences were observed between (3) vs. (1, 2). ^§^ at *p* < 0.05 statistically significant differences were observed between (1) vs. (2, 3, 4).

**Table 4:** Differences in psychosocial characteristics of healthcare professionals by workplace.

	Median (IQR) by Workplace		Difference	95% CI	p * Value
	UHC Osijek (n = 201)	OBC HC (n = 63)			
The ability to perceive and understand emotions	3.7 (3.2–4.1)	3.8 (3.5–4.1)	0.12	−0.06 to 0.35	0.13
The ability to express and label emotions	3.8 (3.3–4.3)	3.9 (3.5–4.1)	0.13	−0.13 to 0.25	0.27
The ability to regulate and manage emotions	3.9 (3.5–4.1)	3.9 (3.6–4.4)	0.10	−0.05 to 0.30	0.12
**Overall emotional competence score (UEK-45)**	3.7 (3.4–4.1)	3.9 (3.6–4.2)	0.13	−0.02 to 0.29	0.11
**Overall mental health (MHI-5)**	22 (18–24)	21 (20–24)	0	−1 to 1	0.89
**Empathy in palliative care**	4.2 (3.8–4.7)	4.6 (4–4.8)	0.31	0.08 to 0.46	**<0.001**
Impossibility of curing cancer	2 (1.8–2.3)	2 (1.5–2)	−0.25	−0.25 to 0	**0.01**
Stereotypes	2.5 (2.3–2.8)	2.5 (2–2.8)	0	0 to 0.25	0.78
Discrimination	1 (1–1.9)	1 (1–1.8)	0	0 to 0	0.23
**Overall cancer-related stigma**	1.9 (1.8–2.2)	1.8 (1.6–2.2)	−0.08	−0.25 to 0	0.11
**Attitudes towards cancer**					
Perceived controllability of cancer	2.6 (2.2–2.8)	2.6 (2.4–2.8)	0	0 to 0.2	0.30
Negative emotional response	2.9 (2.1–3)	3.0 (2.6–3)	0.14	0 to 0.29	0.12
Intention for proactive behaviour	2.6 (2.3–2.9)	2.9 (2.4–3.1)	0.13	0 to 0.38	**0.02**

* Mann–Whitney U test. Bold value denotes statistical significance. Abbreviation: IQR—Interquartile Range.

**Table 5:** Associations between psychosocial characteristics in all respondents and in groups by occupation and workplace.

	Spearman’s Correlation Coefficient Rho (*p*-Value)					
	Emotional Competence Questionnaire (UEK-45)	Overall Mental Health (MHI-5)	Empathy in Palliative Care	Cancer-Related Stigma Questionnaire	Perceived Controllability of Cancer	Negative Emotional Responses
All participants						
Overall mental health (MHI-5)	**0.141 (0.02)**	-				
Empathy in palliative care	**0.506 (<0.001)**	**0.159 (0.01)**	-			
Cancer-related stigma questionnaire	**−0.151 (0.01)**	**−0.183 (<0.001)**	**−0.187 (<0.001)**	-		
Perceived controllability of cancer	0.098 (0.11)	−0.017 (0.78)	0.080 (0.19)	0.075 (0.22)	-	
Negative emotional responses	−0.065 (0.29)	**−0.313 (<0.001)**	−0.062 (0.31)	**0.184 (<0.001)**	**0.126 (0.04)**	-
Intention for proactive behaviour	**0.209 (<0.001)**	−0.017 (0.78)	**0.203 (<0.001)**	0.112 (0.07)	**0.128 (0.04)**	**0.227 (<0.001)**
Physicians						
Overall mental health (MHI-5)	0.111 (0.20)	-				
Empathy in palliative care	**0.532 (<0.001)**	0.152 (0.08)	-			
Cancer-related stigma questionnaire	**−0.203 (0.02)**	**−0.201 (0.02)**	**−0.269 (<0.001)**	-		
Perceived controllability of cancer	0.142 (0.10)	−0.048 (0.58)	**0.217 (0.01)**	0.095 (0.28)	-	
Negative emotional responses	−0.073 (0.40)	**−0.310 (<0.001)**	−0.059 (0.50)	**0.264 (<0.001)**	0.159 (0.07)	-
Intention for proactive behaviour	**0.274 (<0.001)**	0.088 (0.31)	**0.187 (0.03)**	0.060 (0.49)	0.136 (0.12)	**0.188 (0.03)**
Other healthcare professionals						
Overall mental health (MHI-5)	**0.188 (0.03)**	-				
Empathy in palliative care	**0.479 (<0.001)**	0.155 (0.08)	-			
Cancer-related stigma questionnaire	−0.100 (0.26)	−0.165 (0.06)	−0.116 (0.19)	-		
Perceived controllability of cancer	0.048 (0.58)	0.024 (0.78)	−0.034 (0.70)	0.073 (0.41)	-	
Negative emotional responses	−0.071 (0.42)	**−0.332 (<0.001)**	−0.069 (0.44)	0.098 (0.27)	0.074 (0.41)	-
Intention for proactive behaviour	0.148 (0.09)	−0.120 (0.17)	**0.204 (0.02)**	0.158 (0.07)	0.137 (0.12)	**0.268 (<0.001)**
UHC Osijek						
Overall mental health (MHI-5)	0.086 (0.23)	-				
Empathy in palliative care	**0.485 (<0.001)**	0.135 (0.06)	-			
Cancer-related stigma questionnaire	**−0.192 (0.01)**	**−0.219 (<0.001)**	**−0.27 (<0.001)**	-		
Perceived controllability of cancer	0.123 (0.08)	0.022 (0.76)	0.076 (0.28)	0.045 (0.52)	-	
Negative emotional responses	−0.037 (0.60)	**−0.286 (<0.001)**	−0.027 (0.71)	**0.206 (<0.001)**	0.086 (0.22)	-
Intention for proactive behaviour	**0.165 (0.02)**	−0.035 (0.63)	**0.168 (0.02)**	0.087 (0.22)	0.108 (0.13)	**0.241 (<0.001)**
OBC HC						
Overall mental health (MHI-5)	**0.309 (0.01)**	-				
Empathy in palliative care	**0.565 (<0.001)**	**0.310 (0.01)**	-			
Cancer-related stigma questionnaire	−0.038 (0.77)	−0.103 (0.42)	0.140 (0.27)	-		
Perceived controllability of cancer	−0.030 (0.81)	−0.158 (0.22)	0.010 (0.94)	0.201 (0.11)	-	
Negative emotional responses	−0.220 (0.08)	**−0.372 (<0.001)**	**−0.308 (0.01)**	0.199 (0.12)	**0.260 (0.04)**	-
Intention for proactive behaviour	**0.335 (0.01)**	0.039 (0.76)	**0.283 (0.02)**	**0.272 (0.03)**	0.156 (0.22)	0.153 (0.23)

Bold value denotes statistical significance.

**Table 6:** Multivariate linear regression analyses of factors associated with cancer-related stigma and empathy in palliative care among healthcare professionals.

	ß	p * Value	95% CI for ß	Model
Cancer-related stigma				
Empathy in palliative care	−0.094	**0.01**	−0.17 to −0.02	R^2^ = 0.112, R_adj_^2^ = 0.089 F_(7256)_ = 4.6; *p* < 0.001 Cohen’s f^2^ = 0.13
Negative emotional responses	0.084	**0.01**	0.02 to 0.15	
Intention for proactive behaviour	0.096	**0.02**	0.02 to 0.18	
Empathy in palliative care				
Emotional competence (UEK-45)	0.52	**<0.001**	0.40 to 0.64	R^2^ = 0.280, R_adj_^2^ = 0.266 F_(5258)_ = 20.0 *p* < 0.001 Cohen’s f^2^ = 0.39

Bold value denotes statistical significance. Abbreviation: ß—regression coefficient; CI—confidence interval; * Adjusted for gender, age, profession, and workplace.

## Data Availability

All data are available and can be delivered to anyone upon request.

## Abbreviations

The following abbreviations are used in this manuscript:

ß	Regression coefficient
CI	Confidence interval
GM	General medicine
IQR	Interquartile Range
MHI-5	The Mental Health Inventory-5
OBC HC	Osijek-Baranya County Healthcare Centre
UHC Osijek	University Hospital Centre Osijek
UEK-45	The Emotional Competence Questionnaire
